# Enteric Neurospheres Are Not Specific to Neural Crest Cultures: Implications for Neural Stem Cell Therapies

**DOI:** 10.1371/journal.pone.0119467

**Published:** 2015-03-23

**Authors:** Ellen Binder, Dipa Natarajan, Julie Cooper, Rania Kronfli, Mara Cananzi, Jean-Marie Delalande, Conor McCann, Alan J. Burns, Nikhil Thapar

**Affiliations:** 1 Stem Cells and Regenerative Medicine, UCL Institute of Child Health, London, United Kingdom; 2 Department of Clinical Genetics, Erasmus MC, Rotterdam, The Netherlands; 3 Department of Gastroenterology, Great Ormond Street Hospital for Children, London, United Kingdom; University Of Melbourne, AUSTRALIA

## Abstract

**Objectives:**

Enteric neural stem cells provide hope of curative treatment for enteric neuropathies. Current protocols for their harvesting from humans focus on the generation of ‘neurospheres’ from cultures of dissociated gut tissue. The study aims to better understand the derivation, generation and composition of enteric neurospheres.

**Design:**

Gut tissue was obtained from *Wnt1-Cre;Rosa26^Yfp/Yfp^* transgenic mice (constitutively labeled neural crest cells) and paediatric patients. Gut cells were cultured either unsorted (mixed neural crest/non-neural crest), or following FACS selection into neural crest (murine-YFP+ve/human-p75+ve) or non-neural crest (YFP-ve/p75-ve) populations. Cultures and resultant neurospheres were characterized using immunolabelling *in vitro* and following transplantation *in vivo*.

**Results:**

Cultures of (i) unsorted, (ii) neural crest, and (iii) non-neural crest cell populations generated neurospheres similar in numbers, size and morphology. Unsorted neurospheres were highly heterogeneous for neural crest content. Neural crest-derived (YFP+ve/p75+ve) neurospheres contained only neural derivatives (neurons and glia) and were devoid of non-neural cells (i.e. negative for SMA, c-Kit), with the converse true for non-neural crest-derived (YFP-ve/p75-ve) ‘neurospheres’. Under differentiation conditions only YFP+ve cells gave rise to neural derivatives. Both YFP+ve and YFP-ve cells displayed proliferation and spread upon transplantation in vivo, but YFP-ve cells did not locate or integrate within the host ENS.

**Conclusions:**

Spherical accumulations of cells, so-called ‘neurospheres’ forming in cultures of dissociated gut contain variable proportions of neural crest-derived cells. If they are to be used for ENS cell replacement therapy then improved protocols for their generation, including cell selection, should be sought in order to avoid inadvertent transplantation of non-therapeutic, non-ENS cells.

## Introduction

Severe gut motility disorders result from developmental or acquired abnormalities of the enteric nervous system (ENS) and include aganglionosis in Hirschsprung disease (HSCR), dysganglionosis or neuronal dysfunction seen in chronic intestinal pseudo-obstruction (CIPO) and immune-mediated neuronal destruction in conditions such as oesophageal achalasia [1–4]. Current definitive treatment of such motility disorders, especially HSCR, is limited to surgical resection of aganglionic or affected bowel. However, the long-term outcomes of surgery remain poor [5–9] and novel therapies are required.

Over the last few years, several groups have been working towards developing enteric nervous system (ENS) cell replacement therapy strategies, which have been greatly aided by advances in the field of neural stem cell biology [10–13]. Here the hypothesis is that stem cells, capable of generating a sufficient ENS to effect functional recovery when transplanted into the affected guts of patients with HSCR or other ENS disorders, could improve outcomes following surgical treatments, or eliminate the need for surgery altogether [12,14,15].

The persistence in foetal and postnatal life of neural stem cells either from CNS [16] or ENS of mice [17] or human [18] prompted several groups to explore how such cells could be isolated, cultured and ultimately transplanted into either wild type or aganglionic gut [19–21]. In order to maximise the efficiency of replenishing the ENS, and given its entire derivation from neural crest, transplanting a purer population of neural crest-derived cells would seem to be most favourable [22,23]. This ideal requirement appears to have been satisfied when many of the above mentioned studies demonstrated that neural crest cells, that retain characteristics of early ENS progenitors, could be isolated as single cells and cultured to form large cellular aggregates termed neurospheres or neurosphere-like bodies (NLBs). These neurospheres have been shown to form from mouse gut when cultured in enriched medium [17] and also from human gut cultures [18,20,24]. They have been regarded as niches for enteric neural crest stem cells and their formation has been established as an initial method of selecting and enriching for large numbers of neural crest cells in culture, especially from human gut tissue [18,20,24,25].

Thus, much of the enteric neural stem cell transplantation research in mice so far has been performed using neurospheres derived from neural crest cells. In mouse this has been refined by isolating cells using either p75 as a selection marker or from transgenic mouse lines expressing GFP under specific ENS promoters such as Ret, EDNRB or activated by Wnt1-Cre [20,21,25–27]. These authors showed that the transplanted neurosphere-derived cells proliferated and gave rise to glia and neurons, including several neuronal subtypes, some of which exhibited neurochemical and electrophysiological activity similar to endogenous neurons [21].

The positive outcomes of these animal studies have underpinned the drive to translate this research to humans. Towards this, significant progress has been made in the harvesting of ENS stem cells from human gut. We and other groups have reported the isolation of such cells from post-natal human full-thickness gut tissue from resection specimens obtained at surgery or from gut mucosal tissue obtained using conventional, minimally invasive intestinal endoscopic techniques gut [18–20,24,26]. As opposed to the animal studies, however, no published studies of human enteric neural stem cells have, to date, utilized specific selection of enteric neural cells, relying entirely on the generation of neurospheres or NLBs. It is therefore critical that the nature of enteric neurospheres is carefully elucidated prior to delivery into patients. Hence the aim of this study is to better understand the generation and composition of neurospheres that arise in culture of dissociated gut.

## Materials and Methods

### Animals

Mice carrying a conditional *Yfp* allele in the Rosa26 locus (MGI:2449038) [28] were mated to mice expressing Cre recombinase under the control of the Wnt1 promoter/enhancer (MGI:2386570) [29] to obtain *Wnt1-Cre;Rosa26* ^*Yfp/Yfp*^ offspring that express YFP in NCC [30], including enteric NCC [31]. All experiments were conducted in accordance with the UK Home Office regulations for animal experimentation.

### Human tissue

Human postnatal mucosal gut biopsy specimens were obtained from children undergoing colonoscopy at Great Ormond Street Hospital (GOSH), London, UK, under ethical approval following informed consent.

### Isolation and culture of enteric neural crest stem cells

Gastrointestinal tracts were dissected from embryonic (E12.5-E18.5) and postnatal (P0-P8) mice. These mouse tissues, and human postnatal gut tissues were dissociated and cultured as described in the Supplementary Materials (S1 Materials). Neurospheres were generated after a few days up to two weeks in both cultures using methods described in detail in the Supplementary Materials (S1 Materials). For wholemount immunostaining, both mouse and human neurospheres were fixed with 4% PFA in PBS for 15 min.

### Fluorescence-activated cell sorting (FACS)

Gut tissue from *Wnt1-Cre;Rosa26* ^*Yfp/Yfp*^ mice was dissociated as above and resuspended in NSM with 2% fetal bovine serum (FBS, Sigma UK). Cells were sorted with a MoFloXDP cell sorter (Beckman Coulter). The yellow fluorescent protein positive (YFP+ve) cells were selected using a 530/40 filter set. Gating parameters were set using cells from wild-type gut and applied to increase specificity of selection of YFP+ve and YFP negative (YFP-ve) cells. BothYFP+ve and YFP-ve cell populations were collected. These populations, along with an unsorted cell population, were plated separately onto fibronectin-coated dishes.

To isolate neural crest and non-neural crest derived cells from human gut samples, p75^NTR^ conjugated to phycoerythrin (p75PE, Abcam, UK) was used. Cultured cells were dissociated and incubated with the antibody for 1 hour on ice, washed twice with medium and cells subjected to FACS. For human p75PE positive (p75+ve) cell isolation, cells were selected using a 580/30 filter set. Gating parameters were set using unlabelled cells. As above, both p75+ve and p75PE negative (p75-ve) cells were collected and plated onto fibronectin coated dishes.

### Transduction of YFP-ve cells using lentivirus

Following FACS to select YFP+ve cells, the YFP-ve population was transduced with a GFP containing lentivirus following a published protocol [32,33]. Lentivirus was added to cultures at concentrations in the range of 2–5 MOI. Cells were incubated for 24–48 hours to allow cells to be transduced and for viral particles to self-inactivate.

### Transplantation of neurospheres into mouse gut

Neurospheres derived from both YFP+ve and YFP-ve cell cultures were transplanted into the distal colon of mice, exposed by laparotomy, using a pulled glass needle. The peritoneum was closed using absorbable sutures and the skin with wound clips, which were removed after 7 days. The gut from transplanted mice was analysed 1–3 months later.

### Immunolabelling

Following fixation in 4% PFA, cells were pre-incubated for 1h in blocking solution (BS) comprising PBS with 1% BSA, 0.1% Triton X-100 and 0.15% glycine. For neurospheres, blocking solution contained 1% Triton X-100. Primary and secondary antibodies (Table 1) were applied in blocking solution. Cells were incubated in primary antibodies for 1–4h at RT and intact neurospheres were incubated for either 3–5h at RT or overnight at 4°C. Following several washings with PBS + 0.1% Triton X-100 (PBT), cells and neurospheres were incubated in secondary antibodies (Table 1) containing DAPI for 1h at RT. Cells and neurospheres were mounted using either Vectashield HardSet (Vector laboratories) or Aqua-Poly/Mount (Polysciences).

**Table 1:** 

Primary Antibody	Species raised in	Dilution	Source
Anti-Sox10	Goat	1:300	Santa Cruz Biotechnology
anti-p75	Rabbit	1:300	Santa Cruz Biotechnology
anti-Nestin	Mouse	1:50	Millipore
anti-GFP	Mouse	1:300	Life Technologies
anti-GFP	Rabbit	1:300	Life Technologies
anti-GFP	Chicken	1:300	abcam
anti-TuJ1	Mouse	1:500	Covance
anti-TuJ1	Rabbit	1:500	Covance
anti-PGP9.5	Mouse	1:100	abcam
anti-NPY	Rabbit	1:200	Santa Cruz Biotechnology
anti-CGRP	Rabbit	1:100	Santa Cruz Biotechnology
anti-nNos	Rabbit	1:300	Life Technologies
anti-VIP	Rabbit	1:300	AbDSerotec
anti-GFAP	Rabbit	1:500	Dako
anti-S100	Rabbit	1:500	Dako
anti-SMA	Mouse	1:200	Dako
anti-c-Kit	Rabbit	1:50	Dako
anti-Ki67	Rabbit	1:500	Novocastra
anti-phospho Histone H3	Rabbit	1:100	Millipore
Secondary Antibody	Species raised in	Dilution	Source
Alexa fluor anti-mouse 488	Goat	1:500	Life Technologies
Alexa fluor anti-rabbit 488	Goat	1:500	Life Technologies
Alexa fluor anti-mouse 568	Goat	1:500	Life Technologies
Alexa fluor anti-rabbit 568	Goat	1:500	Life Technologies
Biotinylated anti-goat	Rabbit	1:500	Life Technologies
Streptavidin, Alexa fluor 555	N/A	1:500	Life Technologies

Immunolabelled gut samples were analyzed using a Leica SPE1 confocal microscope (Leica Microsystems). Images are displayed as single sections or as a merge of a number of serial sections. Figures were compiled using Adobe Photoshop and Illustrator software.

## Results

### Distribution, selection and proportion of YFP+ve cells in developing mouse gut

**Fig 1:**
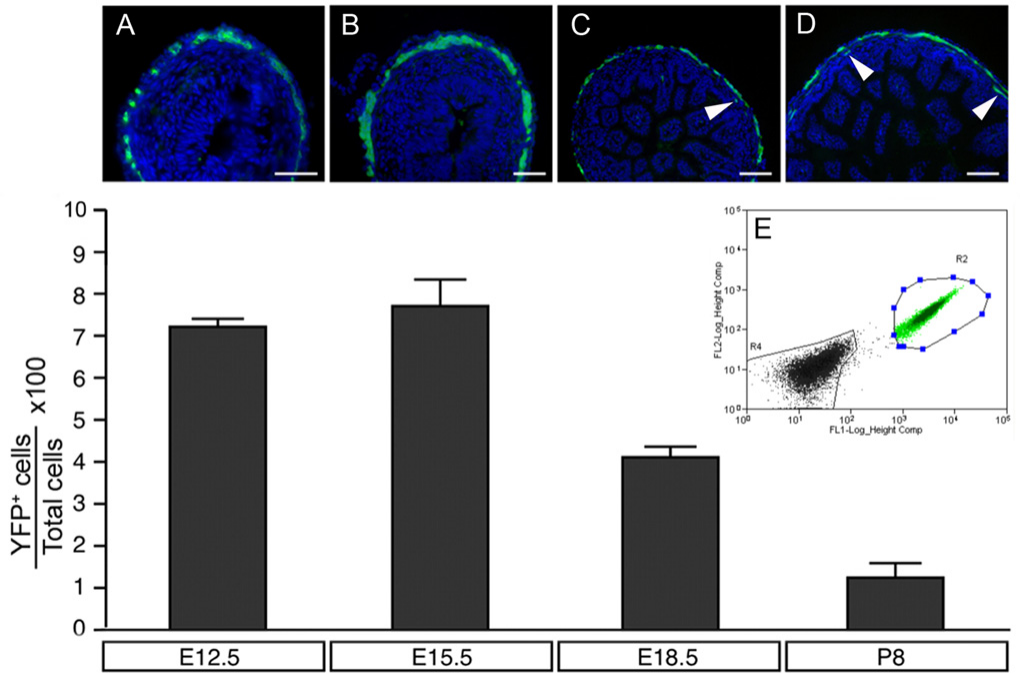
Distribution, selection and proportion of YFP+ve cells in developing mouse gut. (A-D) Transverse sections of *Wnt1-Cre;Rosa26* ^*Yfp/Yfp*^ mouse gut showing distribution of YFP+ve cells at the level of the presumptive myenteric (A-D) and submucosal (C,D arrowheads) plexuses. (E) FACS selection of YFP+ve cells. Bar graph shows the proportion of YFP+ve cells at selected stages of development. Scale bars = 50μm in A,B and 100μm in C,D.

*Wnt1-Cre;Rosa26* ^*Yfp/Yfp*^ mouse gut was harvested at E12.5, E15.5, E18.5 and P8, cryosectioned and immunolabelled with anti-GFP antibody (which also labels YFP cells). YFP+ve cells were located within the outer gut layers, corresponding to the presumptive myenteric plexues of E12.5 and E15.5 mice (Fig. 1A, 1B) and within the myenteric and submucosal plexus layers of E18.5 and P8 mice (Fig. 1C, 1D). Guts from these mice were also dissociated and sorted using FACS for YFP (Fig. 1E). At E12.5, 7.1±0,25% (n = 3) of all cells analysed were YFP+ve. The highest proportion of YFP+ve cells, 7.68%±0.62% (n = 5), was detected at E15.5. At later stages (E18.5 and P8) the percentage of YFP+ve cells decreased to 4.13±0.22% (n = 6) and 1.22±0.38% (n = 4) respectively (Fig. 1A). We used guts from E15.5 mice since they had the highest proportion of YFP+ve cells.

### Neurospheres arise from neural crest and non-neural crest-derived gut cell populations

**Fig 2:**
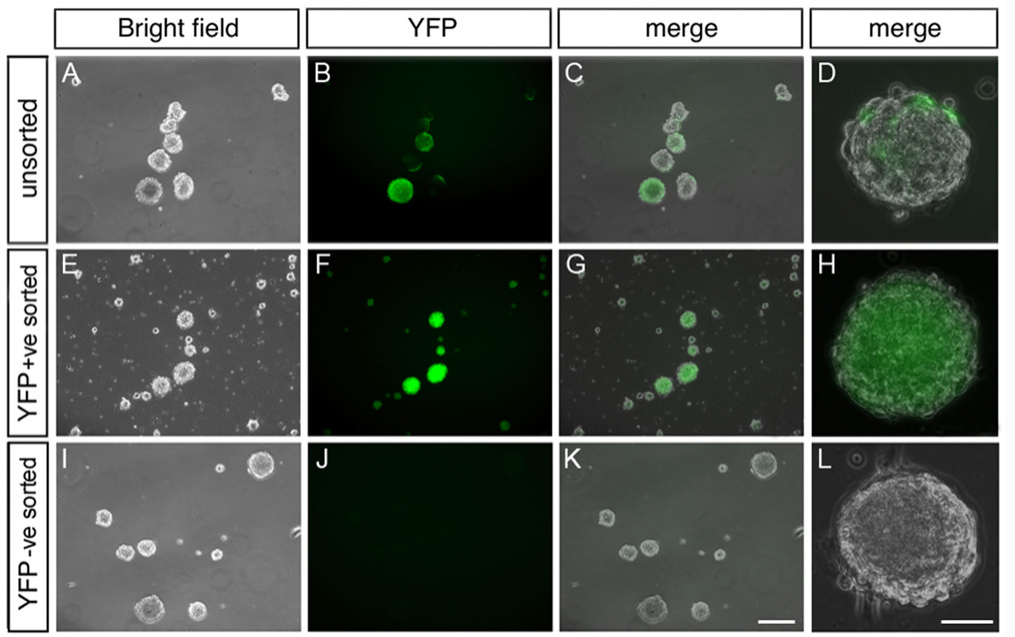
Neurospheres can be generated from YFP+ve, YFP-ve and mixed gut cell populations. Panels show phase contrast images of cultures containing neurospheres and their YFP fluorescent counterparts. (A-D) Neurospheres from unsorted gut cells contain both YFP+ve and YFP-ve cells whereas neurospheres derived from YFP+ve sorted cells (E-H) contain only YFP+ve cells. (I-L) Neurospheres from cultures of YFP-ve, non-neural crest cells do not contain YFP+ve cells. Neurospheres are similar in size and appearance in all cases. Scale bar in G = 200μm (applies to A-C, E-G, I-K) and in H = 40μm (applies to D, H, L).

In order to study neurosphere generation, we used cultures containing cells from three sources; *Wnt1-Cre;Rosa26* ^*Yfp/Yfp*^ mouse gut tissue to segregate, by FACS, (i) YFP+ve neural crest-derived cells from (ii) YFP-ve non-neural crest-derived cells, while also retaining a pool of (iii) unsorted gut-derived cells. After being maintained in optimal culture conditions for up to 7 days, neurospheres formed in all three groups of cells (Fig. 2). Unsorted cells gave rise to neurospheres containing both YFP+ve fluorescent cells and YFP-ve cells (Fig. 2A-2D). The neurospheres that arose from the YFP+ve cells contained only fluorescent cells (Fig. 2E-2H) whereas those from YFP-ve cells did not contain any fluorescent cells (Fig. 2I-2L). Details of neurospheres generated were as follows: Average number of neurospheres per well; unsorted = 121±21.7, YFP+ve = 99±1.8, YFP-ve = 129.8±13.8, N = 4 experiments; Average size of neurospheres per well: unsorted = 134.6±46μm, YFP+ve = 137.85±50μm YFP-ve = 105±30μm, N = 4 experiments. The morphology of the neurospheres was comparable between the three groups. These findings suggest that the phenomenon of neurosphere formation in culture is not restricted to neural crest cells alone.

**Fig 3:**
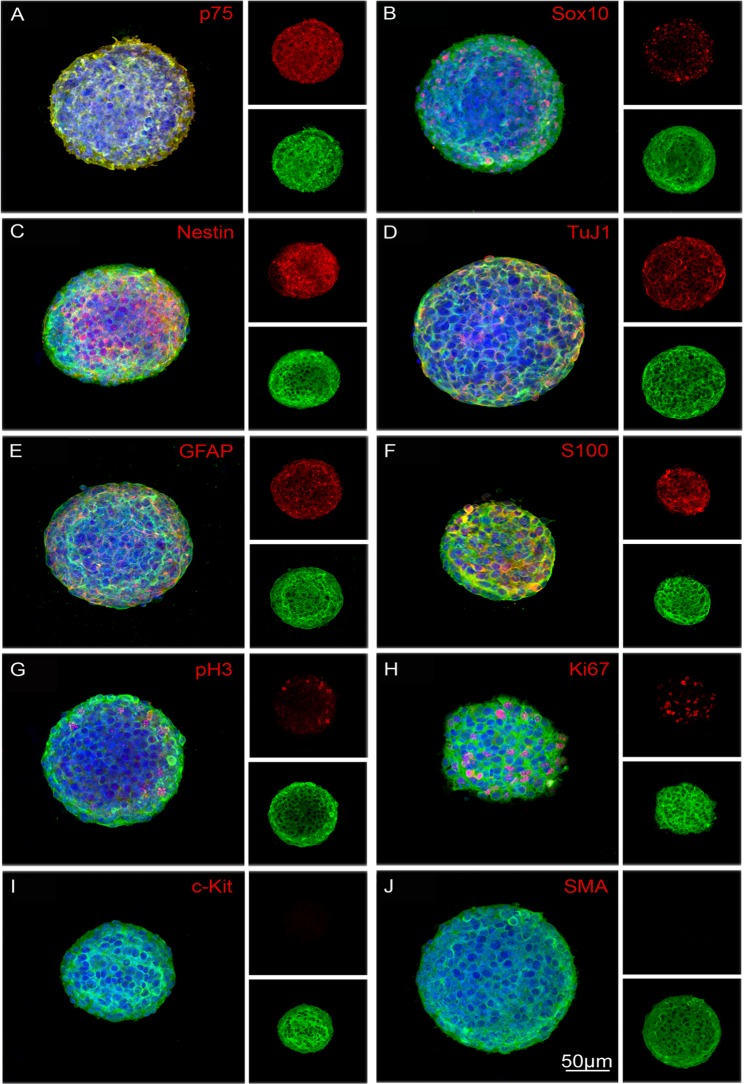
Immunocytochemical characterization of neurospheres generated from sorted YFP+ve neural crest-derived cells. YFP+ve neurospheres contain cells positive for neural crest and neural stem/progenitor markers p75 (A), Sox10 (B) and Nestin (C), the neuronal marker TuJ1 (D), glial markers GFAP (E) and S100 (F), and the proliferation markers PH3 (G) and Ki67 (H, arrows). YFP+ve neurospheres do not contain cells expressing the ICC marker c-Kit (I) or the smooth muscle marker, SMA (J). All neurospheres show YFP+ve cells in green, corresponding markers in red and DAPI (nuclei) in blue. Scale bar = 50μm.

**Fig 4:**
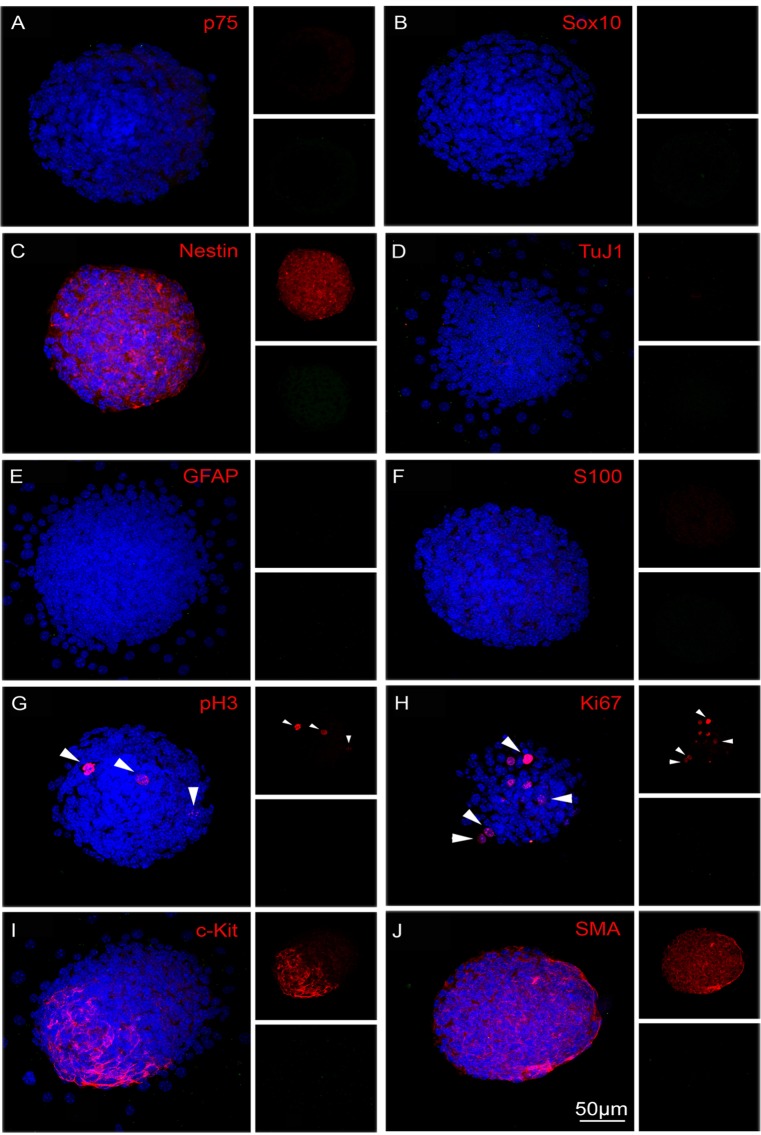
Characterization of neurospheres generated from YFP-ve, non-neural crest-derived cells. Neurospheres derived from YFP-ve cells do not contain cells expressing neural crest cell markers p75 (A), Sox10 (B), neuronal marker TuJ1 (D) or glial markers GFAP (E) or S100 (F). However they do contain cells expressing Nestin (C), proliferation markers PH3 (G, arrows), Ki67 (H, arrows), ICC marker c-Kit (I) and smooth muscle marker SMA (J). All markers in red and DAPI (nuclei) in blue. Scale bar = 50μm.

We next characterised, by immunolabelling, the composition of YFP+ve and YFP-ve neurospheres (Fig. 3). Intact YFP+ve neurospheres contained cells that were positive for the neural progenitor cell markers p75, Sox10 and Nestin (Fig. 3A-3C), the pan neuronal marker TuJ1 (Fig. 3D), and the glial markers GFAP and S100 (Fig. 3E, 3F). Moreover, some cells were labelled with PH3 and Ki67 (Fig. 3G and 3H) indicating that they were undergoing proliferation. No cells within these neurospheres were positive for either the ICC marker c-Kit or the smooth muscle actin marker SMA (Fig. 3I and 3J respectively). In contrast, YFP-ve neurospheres were negative for neural progenitor/neural crest cell markers p75 and Sox10 (i.e. confirming absence of neural crest-derived cells), although Nestin expressing cells were present (Fig. 4A-4C). These neurospheres were also negative for the neural and glial cell markers (Fig. 4D-4F). Some cells within the YFP-ve neurospheres were proliferating as shown by PH3 and Ki67 expression (Fig. 4G, 4H), and some were immunopositive for c-Kit and SMA (Fig. 4I, 4J respectively).

**Fig 5:**
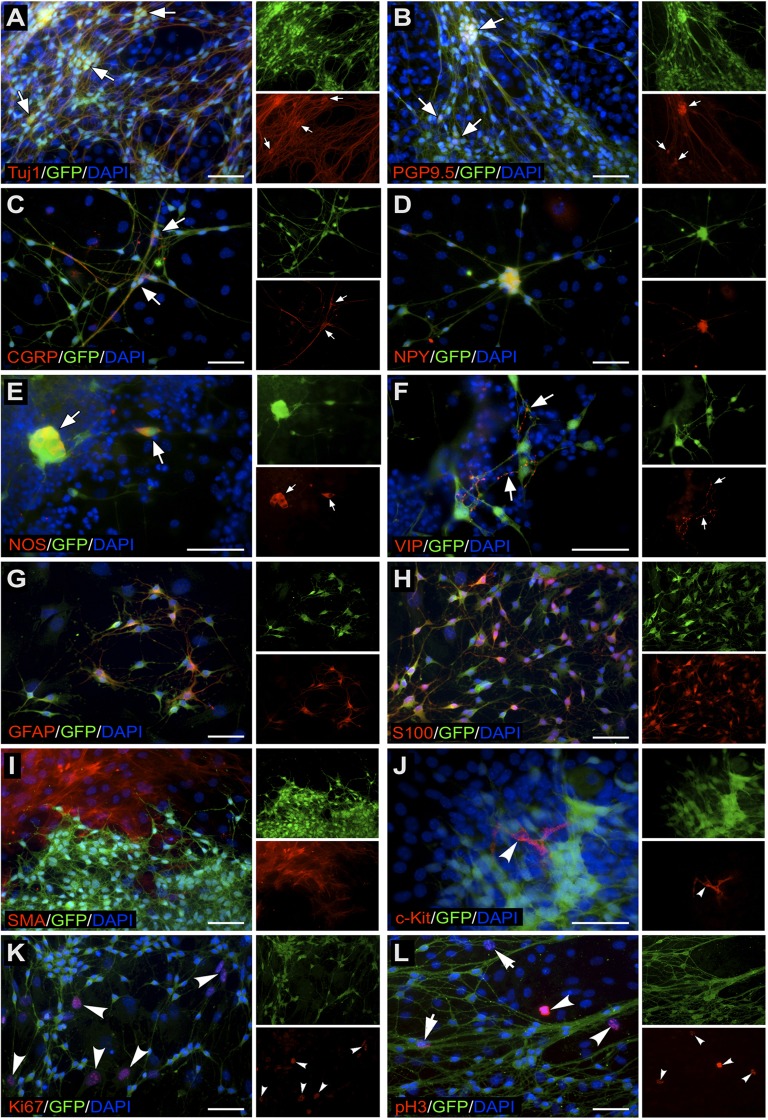
Phenotypes of YFP+ve and YFP-ve cells derived from mixed population neurospheres. YFP+ve cells co-express the pan-neuronal markers TuJ1 (A) and PGP9.5 (B), neuronal subtype markers CGRP (C), NPY (D), nNOS (E), VIP (F), and the glial cell markers GFAP (G) and S100 (H). YFP-ve cells derived from mixed population neurospheres immunolabel with non-neural crest lineage markers. YFP-ve cells are positive for SMA (I) and for c-Kit (J), and some cells co-express the proliferation markers Ki67 (K, arrowheads) and PH3 (L, arrowheads). Arrows in (L) indicate proliferating YFP+ve cells. DAPI (blue) labels nuclei. Scale bar = 50μm.

In order to assess the fates of YFP+ve and YFP-ve cells that comprise mixed population neurospheres, neurospheres generated from unsorted gut cells were cultured under differentiation conditions for 10 days. Within the mixed cell population, YFP+ve cells exclusively co-immunolabelled with a variety of neuronal and neuronal subtype markers, including TuJ1, PGP9.5, CGRP, NPY, nNOS and VIP (Fig. 5A-5F). They also co-labelled with the glial cell markers S100 and GFAP (Fig. 5G, 5H). However they did not differentiate into non-neural crest derivatives such as ICC or smooth muscle cells. In contrast, under the same culture conditions, YFP-ve cells within the mixed population labelled with SMA (Fig. 5I) and c-Kit (Fig. 5J), and not with any of the markers of neural crest cell derivatives. Both YFP+ve (Fig. 5L, arrows) and YFP-ve cells (Fig. 5K, 5L arrowheads) expressed the proliferation markers PH3 and Ki67. Thus, although both neural crest and non-neural crest cell populations are capable of forming neurospheres, the cell phenotypes that comprise these spheres are different and remain distinct from each other.

### YFP+ve cells associate with host ENS following transplantation

**Fig 6:**
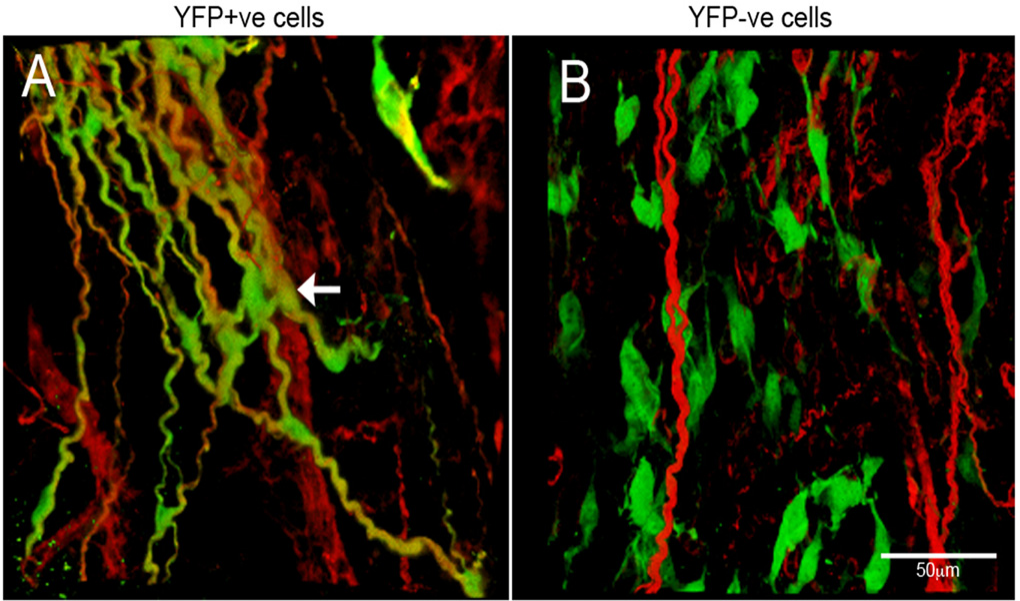
Transplantation of neurospheres into mouse gut. (A) Confocal images of host gut transplanted with YFP+ve neurospheres. YFP+ve cells spread from the site of transplantation and integrated with the endogenous ENS as shown by TuJ1 immunolabelling (red). (B) Host gut transplanted with YFP-ve neurospheres. YFP-ve cells, labelled with lentiviral construct expressing GFP (green), did not integrate with the endogenous ENS (red) and were apparent as individual, isolated cells. Scale bar = 50μm.

In order to assess the ability of YFP+ve and YFP-ve cells to associate with and integrate into recipient ENS, neurospheres from both sources were transplanted into the distal colon of wild type mice and analysed 3 to 6 months later. Cells from YFP+ve neurospheres spread out, localised within the myenteric and submucosal plexuses, and integrated within the endogenous ENS (Fig. 6A). YFP+ve cells co-expressed the neuronal marker TuJ1 and extended extensive projections that intermingled with the endogenous ENS network (Fig. 6A, arrow). YFP-ve cells (which were transduced with a GFP containing lentivirus to fluorescently label them for identification post transplantation) also spread out, but they were separate from each other, and were not specifically located with or integrated within the ENS layers, and they did not co-express TuJ1 (Fig. 6B).

### Neurospheres from human gut tissues contain neural crest and non-neural crest-derived cells

**Fig 7:**
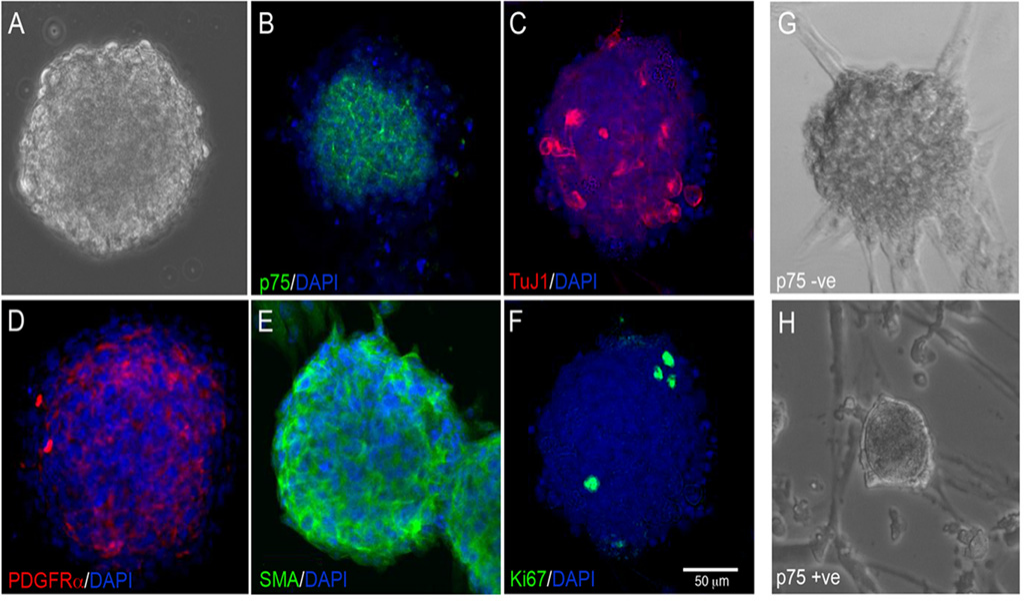
Neurospheres derived from postnatal human gut cultures are heterogeneous in composition. (A) Bright field image of neurosphere derived from human postnatal gut cultured for 2 weeks. Neurospheres contain neural crest-derived cells as shown by expression of the neural crest cell marker p75 (B) and the neuronal marker TuJ1 (C). Neurospheres also contained non-neural crest cell derivatives such as fibroblast-like cells expressing PDGFRα (D), and muscle cells expressing SMA (E). Proliferating cells are labelled with Ki67 (F). Following FACS sorting based on selection with p75^NTR^, neurospheres were generated exclusively from p75-ve (G) and p75+ve cells (H) similar in shape and appearance. DAPI (blue) stains nuclei in B-F. Scale bar = 50μm.

Postnatal human gut tissue was dissociated, cultured, ± sorted with p75 to form neurospheres by day 14 (Fig. 7A). Although the unsorted and sorted neurospheres were similar in appearance and shape to those derived from mouse gut, they were generated in fewer numbers (Average number of neurospheres per well; unsorted = 23.7±2.7, p75PE+ve = 4.8±0.8, p75PE-ve = 17.6±4.5 N = 3 experiments) and were slightly smaller (Average size of neurospheres per well; unsorted = 90.6±13.9μm, p75PE+ve = 79.3±13.7μm p75PE-ve = 103±10.4μm; N = 3 experiments). Immunohistochemical analysis revealed that unsorted human neurospheres contained neural crest-derived cells such as ENS progenitors labelled with p75 (Fig. 7B) and enteric neurons, as shown by labelling with TuJ1 (TuJ1 = 4.1±2.3%) (Fig. 7C), as well as non-neural crest cell derivatives such as fibroblast-like cells (PDGFRα, Fig. 7D) and smooth muscle cells (SMA, Fig. 7E). They also contained proliferating cells that labelled with Ki67 (Fig. 7F). Neural crest and non-neural crest-derived cells were sorted by FACS, based on the expression of the neural crest-specific marker p75. Following cell culture, the resulting neurospheres generated from p75-ve cells (Fig. 7G) and p75+ve cells (Fig. 7H) were similar in size and appearance to each other, and to mixed population neurospheres (Fig. 7A). The p75+ve sorted neurospheres were made up almost exclusively of neurons and glia (TuJ1 = 74.3±25%; S100 = 23.6±2%). Thus, like mouse gut-derived neurospheres, human neurospheres contain a heterogeneous mix of neural crest and non-neural crest-derived cells. These can be enriched for neural crest-derived cells by sorting with a marker such as p75.

## Discussion

Current opinion appears to suggest that gut-derived neurospheres represent therapeutic packages for the treatment of severe gastrointestinal neuropathies. Here we confirm that neurospheres can be generated in cultures of dissociated embryonic and post-natal mouse gut, and from human gut tissues. However, using cell selection specifically for neural crest cells, we show that so-called enteric ‘neurospheres’ can also form from non-neural crest populations and these structures are visually indistinguishable from those generated exclusively from neural crest cells. As opposed to *bona fide* neural crest-derived neurospheres that exclusively comprise neural cells (neurons, glia, ENS progenitors), those derived from non-neural crest cells never contain such cell types. The latter comprise mesenchymal derivatives (smooth muscle, ICC) capable of proliferation and survival upon transplantation but fail to integrate with the recipient ENS.

Our studies confirm the neural crest origin of neurons and glia and suggest that unlike other postmigratory neural crest cells [34] those present within the intestine are unable to generate non-neural cell types such as smooth muscle even under differentiation conditions. In differentiation cultures, neural cell types were exclusively generated from *Wnt1-Cre;Rosa26* ^*Yfp/Yfp*^-derived YFP+ve cells and non-neural cells only derived from YFP-ve cells. This is contrary to data from Bixby et al. that showed that p75+α4+ cells, selected from post-natal gut, were capable of generating smooth muscle actin positive myofibroblasts in subclonal cultures [35]. In our experiments, neurospheres generated fromp75+ve cells were almost exclusively comprised of neurons and glia, and did not contain smooth muscle positive cells. The specificity of p75 as a marker of NCC is further supported by human data from one of the current authors [36] which showed expression limited to cells in the myenteric and submucosal plexuses. Our findings, consistent with other studies [37,38] also suggest that nestin expression is not specific to neural crest given its extensive expression in both neural crest-derived neurospheres and non-neural crest-derived ‘neurospheres’.

In our human studies it is possible that the high proportion of non-neural crest cells reflected their derivation from the submucosa rather than the myenteric plexus. Becker et al. working with murine gut, showed that both the ability to generate neurosphere-like bodies (NLBS) and the proportions of neural crest and non-neural crest cells contained within them varied according to whether cultures were established from the submucosal or the myenteric plexus [39]. NLBs from the latter were composed predominantly of neural crest-derived cells (expression of *Wnt1-cre*:*tdTomato*), produced in larger numbers, demonstrated greater proliferation and contained higher proportion of neural derivatives than submucosal NLBs, which contained higher numbers of non-neural crest cells (CD34+ and smooth muscle-like cells). In keeping with our findings submucosal NLBs were also seen to form in the absence of neural crest cells [39]. Although we did not carry out a comparison of neurospheres generated from human gut mucosal biopsies with those from full thickness biopsies, our murine and human studies, taken together, suggest that currently accepted protocols to generate enteric neural crest stem cells without cell selection result in hybrid or neural crest devoid neurospheres. Hence, consideration in terms of suitability for therapy should be given. We do show, for the first time, that it is possible to select neural crest cells from human gut mucosal biopsies to generate *bona fide* neurospheres containing neural elements.

The ability to select and enrich for neural crest cells may be important for potential cell transplantation therapies aimed at rescuing or restoring the ENS, which is entirely derived from the neural crest. However, it is still unclear whether it is beneficial to transplant entire neurospheres or pure populations of neural crest cells into aganglionic gut. Neurospheres may represent organized stem cell niches for neural progenitors maintained by differentiated neural and non-neural cells. Theocharatos et al. [40] suggest that neurospheres may encompass a degree of spatial organization of proliferating cells, with a predominance of dividing cells in the periphery. However, our current and previous studies [17,20] do not suggest that neurospheres have a particular organization and confirm that the presence of neural crest cells is not necessary for them to form. It is possible, however, that factors produced by the non-neural crest cells within mixed population neurospheres may provide paracrine factors necessary for neural crest-derived cells to survive, proliferate, integrate and form a functional ENS. It is well established that during embryonic development of the ENS, key mesenchyme-derived factors, such as GDNF and endothelin-3, are required by the migrating neural crest population in order to complete gut colonization and ultimately give rise to the ENS [41–45]. In the adult gut, enteric neuron survival and neurogenesis appears dependent upon 5-HT4 [46]. Further supporting this idea, the successful transplantation of mixed population neurospheres into gut has already been reported. Several groups have shown that neurospheres containing mixed cell populations, when transplanted into aganglionic gut, gave rise to an ENS network containing neurons, glial cells and progenitor cells [18,20,24,26]. Lindley et al. [25] demonstrated that mixed neurospheres transplanted into *in vitro* aganglionic gut regulated functional activity.

Most recently Hetz et al. [24] used human enteric neurospheres generated from postnatal and adult human intestine for *in vivo* transplantation into a mouse model of acquired intestinal hypo/aganglionosis where the intestine was treated prior to transplantation with benzalkonium chloride (BAC), an established means of inducing denervation of the myenteric plexus. The authors reported functional rescue of the BAC treated gut that had been transplanted with cells from enteric neurospheres compared to non-transplanted BAC-treated ‘control’ gut. The mechanism of this recovery, however, was unclear. The authors acknowledge the presence or generation of non-neural crest cells, which in light of our findings may be a considerable proportion given that most donors were beyond the 5^th^ decade of life where neurogenesis is diminished [20,47]. Hetz et al. concluded, from their pilot study, that it was not clear whether the recovery was caused by integration of donor functional neuronal cells or by ‘bystander mechanisms’ [24]. Indeed such positive ‘bystander’ roles have been implicated following the transplantation of mesenchymal, including intestinal myofibroblasts, and other stem cells into models of intestinal inflammation [48,49]. Hetz et al. showed the presence of transplanted alpha-SMA positive cells, which could be myofibroblasts. We know from our studies that intestinal myofibroblasts are likely to comprise a sufficient proportion of the neurosphere cell population, as shown by both the PDGFRα and SMA positive cells. It has been established that the endogenous ENS has the inherent ability to regenerate after BAC treatment. Hanani et al. showed that the endogenous ENS appears capable of regeneration within 7 days of BAC treatment [50]. It is very feasible, therefore, that transplanted human cells in the study by Hetz et al. may have promoted such regeneration and recovery of endogenous neuronal cells in the recipient BAC-treated gut rather than functionally integrating themselves [24]. Although this healing mechanism may be useful to promote ‘healing’ in inflamed gut it will not prove helpful in HSCR or other conditions characterized by neuronal deficiency or dysfunction.

Even though these mixed neurosphere transplantation experiments appear to have been successful, at least in terms of ENS rescue, there may be concomitant negative effects from adding mesenchymal cells, including fibroblasts, into recipient gut. These include long-term safety issues, and excessive scarring or contraction at the site of transplantation. We show that non-neural crest cells are capable of proliferation and upon transplantation spread out but unlike neural crest-derived donor cells fail to locate or integrate within the host ENS. We did not ascertain whether such transplanted cells, upon prolonged follow-up, have malignant potential. Our own observations from *in vivo* transplantation of mixed neurospheres show scarring at the transplantation site (data not shown). Transplantation of other non-neural crest cells may also carry risks including the inadvertent transfer of immune cells capable of causing graft versus host disease. Although it could be argued that the numbers of non-neural crest cells within mixed neurospheres transplanted into recipient gut could be small (1–5% [17]), we show here that the proportion of non-neural crest cells within neurospheres may actually be considerably higher, and even exclusive (Fig. 2). Thus the potentially detrimental effects of transplanting non-neural crest cells into gut may in fact be highly relevant.

Is the transplantation of pure populations of neural crest-derived cells feasible? In the context of transplantation into certain models of ENS disease, the transplantation of pure populations of neural cells may be possible given that the recipient environment is likely to be supportive. For example in Ret mutant mice the defect is cell autonomous for neural crest, and the local gut environment appears to be normal [51]. *RET* mutations comprise the most common encountered in human Hirschsprung disease (reviewed in [52]) again suggesting that in a significant proportion of HSCR patients the recipient gut environment should be favourable for selected neural crest-derived stem cell transplantation. Hotta et al. demonstrated that cells, derived from mouse and selected by virtue of their expression of engineered neural crest markers (*Ednrb* ^*Kik*^ and *Ret* ^*TGM*^), survived upon transplantation into wild-type and aganglionic gut, where they migrated and showed evidence of functional integration [21]. Our experiments not only showed that selection of a pure neural crest population from mouse gut and transplantation to generate neural cell types *in vivo* is possible but that a similar concept is practically feasible from human gut using markers such as p75. Although it is not known whether p75 labels all neural crest cells it appears to be specific for this cell type. Further work needs to address whether it optimally and reliably selects proliferating neural progenitors for therapy. The ability to isolate gut-derived neural crest cells also facilitates the potential of gene therapy once better genetic elucidation of enteric neuropathies is established.

Enteric ‘neurospheres’ generated directly from dissociated gut tissue using contemporary protocols for the purposes of therapy are heterogenous structures, that potentially vary in their content of neural-crest and non-neural crest populations and can result from pure populations of the latter. This heterogeneity is likely to arise from the diverse cell types present within the gut and by an absence of specific cell type selection prior to the establishment of cell cultures. Of course in the Wnt1-Cre;YFP transgenic mice neural crest cell selection is facilitated by the endogenous YFP expression of such cell types. p75 is used in a similar way in the context of human gut samples. There is a possibility that additional factors, such as the stage of development at which the gut is sampled and the medium used to culture the cells, may also influence the diversity of cell types maintained in cell culture and ultimately present within neurospheres. However, in our unpublished studies we did not find a discernable difference in heterogeneity between neurospheres generated from E12.5 to E15.5 unsorted gut cultures. Furthermore, we compared equivalent cultures maintained in a diversity of media including the originally used neural crest media [17] and one that we have used in this study and a previously published investigation. [20]. We did not find differences either in the efficiency of generating neurospheres, or in the heterogeneity of cells contained within them, between these media. With this in mind, and given that our ultimate goal is to apply ENS stem cells for human therapy, we opted to use the simplest media.

This heterogeneous content, however, is not reflected in the visual characteristics of the neurospheres, which appear similar, and suggest that improved or adapted protocols are required to select or generate ‘optimal’ neurospheres for therapy and reduce potential risks of inadvertently transplanting unwanted cell types. Cell selection of neural crest cells from human gut is feasible but needs further elucidation as to its necessity and practicality. These findings are likely to have implications for enteric neural stem cell therapy.

## Supporting Information

S1 Materials Isolation and culture of enteric neural crest stem cells. (DOCX)
