# Supplementary material for: Enteric Neurospheres Are Not Specific to Neural Crest Cultures: Implications for Neural Stem Cell Therapies
Source: PLoS One. 2015 Mar 23;10(3):e0119467. doi: 10.1371/journal.pone.0119467 (PMC4370605; doi:10.1371/journal.pone.0119467)
Supplement: S1 Materials — (DOCX) [file pone.0119467.s001.docx]

*Isolation and culture of enteric neural crest stem cells*

Gastrointestinal tracts were dissected from embryonic (E12.5-E18.5) and postnatal (P0-P8) mice, and washed in calcium and magnesium-free DPBS (Invitrogen, UK) containing 1% penicillin/streptomycin (P/S, Invitrogen, UK). Gut tissue was digested in an enzyme mixture containing 600U/ml Collagenase XI (Sigma-Aldrich), 0.4U/ml Dispase (Roche) and 1% P/S in PBS at 37°C for 10-40 minutes depending on age of gut tissue followed by mechanical trituration with a fire-polished Pasteur pipette and subsequent filtration through a 40µm cell strainer. Unsorted cells (mixed YFP+ve/YFP-ve obtained directly from dissociated gut) or FACS sorted cells (YFP+ve or YFP-ve) were then plated into individual wells (6 well dishes; Nunc, UK) at a density of 2x10^5^ cells per well. These were maintained in culture until neurospheres are generated.

The cells were cultured in serum-free medium (DMEM/F12 (Sigma-Aldrich, UK) containing 20ng/ml bFGF (Peprotech, UK), 20ng/ml EGF (Peprotech UK), 0.5U/ml Heparin (Sigma-Aldrich), N2 supplement (Invitrogen, UK), B27 supplement (Invitrogen, UK) and 1% P/S (Invitrogen, UK) named NSM. For postnatal tissue, 1µg/ml Fungizone was added to the medium. After a fixed time period the cultures were assessed for formation, numbers of neurospheres per well (efficiency of generation), as well as neurosphere size and heterogeneity.

Human postnatal gut tissue was washed with DPBS, chopped into small pieces and digested in 1mg/ml Collagenase Type I (Sigma, UK) for 40 minutes at 37^o^C. After 2 washes with NSM, samples were passed through a 100µ then 40µ cell strainer. Unsorted cells (mixed p75+ve and p75-ve cells) or FACS sorted cells (p75+ve or p75-ve cells) were then plated into individual wells (2% fibronectin-coated 12 well dishes; Nunc, UK) at a density of 5x10^3^ cells per well. These were maintained in culture until neurospheres were generated. Medium used to culture cells was as above except that P/S was replaced with 100µg/ml Primocin (Invitrogen, UK). After a fixed time period the cultures were assessed for formation, numbers of neurospheres per well (efficiency of generation) and neurosphere size.

Mouse neurospheres were passaged by incubation in Accutase (PAA Laboratories GmbH) for 30-45 minutes, followed by dissociation into single cells and re-plating for secondary neurosphere formation. For *in vitro* cell fate analysis, mouse neurospheres were placed in differentiation conditions (NSM without growth factors on 24-well plates containing cover glasses coated with fibronectin (Sigma-Aldrich, UK). After 5-10 days of differentiation, cells were fixed for 10 minutes using 4% paraformaldehyde (PFA) in PBS. For wholemount immunostaining, both mouse and human neurospheres were fixed with 4% PFA in PBS for 15 min and processed for immunostaining.
